# Activation of the *Nkx2.5–Calr–p53* signaling pathway by hyperglycemia induces cardiac remodeling and dysfunction in adult zebrafish

**DOI:** 10.1242/dmm.026781

**Published:** 2017-10-01

**Authors:** Yanyi Sun, Qiuyun Wang, Yuehua Fang, Chunfang Wu, Guoping Lu, Zhenyue Chen

**Affiliations:** Department of Cardiology, Ruijin Hospital, Shanghai Jiaotong University School of Medicine, 197, Ruijin Er Road, Shanghai, 200025, China

**Keywords:** Zebrafish, Apoptosis, Cardiomyopathy, Echocardiography, Hyperglycemia, *Nkx2.5–Calr–p53*, Diabetes

## Abstract

Hyperglycemia is an independent risk factor for diabetic cardiomyopathy in humans; however, the underlying mechanisms have not been thoroughly elucidated. Zebrafish (*Danio rerio*) was used in this study as a novel vertebrate model to explore the signaling pathways of human adult cardiomyopathy. Hyperglycemia was induced by alternately immersing adult zebrafish in a glucose solution or water. The hyperglycemic fish gradually exhibited some hallmarks of cardiomyopathy such as myocardial hypertrophy and apoptosis, myofibril loss, fetal gene reactivation, and severe arrhythmia. Echocardiography of the glucose-treated fish demonstrated diastolic dysfunction at an early stage and systolic dysfunction at a later stage, consistent with what is observed in diabetic patients. Enlarged hearts with decreased myocardial density, accompanied by decompensated cardiac function, indicated that apoptosis was critical in the pathological process. Significant upregulation of the expression of *Nkx2.5* and its downstream targets calreticulin (*Calr*) and *p53* was noted in the glucose-treated fish. High-glucose stimulation *in vitro* evoked marked apoptosis of primary cardiomyocytes, which was rescued by the p53 inhibitor pifithrin-μ. *In vitro* experiments were performed using compound treatment and genetically via cell infection. Genetically, knockout of *Nkx2.5* induced decreased expression of *Nkx2.5*, *Calr* and *p53*. Upregulation of *Calr* resulted in increased *p53* expression, whereas the level of *Nkx2.5* remained unchanged. An adult zebrafish model of hyperglycemia-induced cardiomyopathy was successfully established. Hyperglycemia-induced myocardial apoptosis was mediated, at least in part, by activation of the *Nkx2.5*–*Calr*–*p53* pathway *in vivo*, resulting in cardiac dysfunction and hyperglycemia-induced cardiomyopathy.

## Abstract (precis)

**Summary:** Induction of hyperglycemia gives rise to cardiac apoptosis and dysfunction in adult zebrafish via the *Nkx2.5–Calr–p53* signaling pathway, resulting in adult cardiomyopathy.

## INTRODUCTION

Diabetes mellitus has become a serious threat to public health in the 21st century. It is estimated that the number of adults diagnosed with diabetes worldwide will increase to 300 million by 2025 (Boudina and Abel, 2007; King et al., 1998). Patients with diabetes are at a higher risk of cardiovascular diseases, which have become the major cause of mortality in the diabetic population (Boudina and Abel, 2007; Garcia et al., 1974). Forty years ago, Rubler et al. (1972) first described diabetic cardiomyopathy, which could eventually develop in diabetic patients in the absence of any vascular pathogenesis (Huynh et al., 2014; Cai et al., 2002). Many studies have shown that hyperglycemia is an independent risk factor for cardiac damage, leading to diabetic cardiomyopathy (Cai et al., 2002; Devereux et al., 2000; Singh et al., 2000). Moreover, accumulating evidence has shown increased apoptosis in the hearts of diabetic patients (Cai et al., 2002; Palmieri et al., 2008; Frustaci et al., 2000) and streptozotocin-treated animals (Cai et al., 2002; Kajstura et al., 2001), and indicated its important role in the development of diabetic cardiomyopathy (Huynh et al., 2014, 2013).

Zebrafish (*Danio rerio*) is a well-recognized vertebrate for studying cardiogenesis and human genetic diseases. However, adult zebrafish are still underutilized to model human diseases such as cardiomyopathy and heart failure. Currently, only two adult zebrafish models of cardiomyopathy have been developed (Sun et al., 2009; Ding et al., 2011). Many studies with zebrafish have been performed for examining diabetes (Gleeson et al., 2007; Jiménez-Amilburu et al., 2015; Liang et al., 2000), but the effects of hyperglycemia on the heart of adult zebrafish have never been reported. Thus, the aim of the present study was to characterize the mechanism of cardiac remodeling due to hyperglycemia in an adult zebrafish diabetic model.

*Nkx2.5*, a cardiac homeobox transcription factor, is critical in the generation of cardiac hypertrophy (Akazawa and Komuro, 2005). *Calr*, known as a major Ca^2+^-binding chaperone residing in the endoplasmic reticulum, is an important cardiac embryonic gene (Qiu and Michalak, 2009). Fetal gene reactivation is one of the characteristic genetic alterations involved in the pathology of cardiovascular diseases. *Calr* upregulation induces dilated cardiomyopathy and heart failure in the hearts of adult transgenic mice (Lee et al., 2013). Furthermore, *Calr*-deficient cells exhibit impaired *p53* expression (Mesaeli and Phillipson, 2004). Transcription factor p53, located in the cell nucleus, not only regulates cell cycle progression but also can induce apoptosis in a variety of cells (Allen et al., 2005). Fiordaliso et al. (2001) first proposed the role of p53 in high-glucose-induced ventricular myocyte apoptosis.

*Nkx2.5*, *Calr* and *p53* are important factors in cardiovascular diseases; however, their regulatory effects in the progression of cardiomyopathy have not been extensively studied. A hyperglycemia-induced cardiomyopathy model was developed in adult zebrafish in this novel study. The significant loss of myocardial fibers and impaired cardiac function were noted, suggesting that myocardial apoptosis is a profound cardiac remodeling mechanism involved in the pathogenesis of diabetic cardiomyopathy. Moreover, the *Nkx2.5*–*Calr*–*p53* signaling pathway and its effects on cardiac apoptosis were illustrated in this model and studied via *in vitro* experiments on primary cultured cardiomyocytes (CMs) in adult zebrafish.

## RESULTS

### Induction of hyperglycemia led to alterations of metabolism

**Fig. 1.**
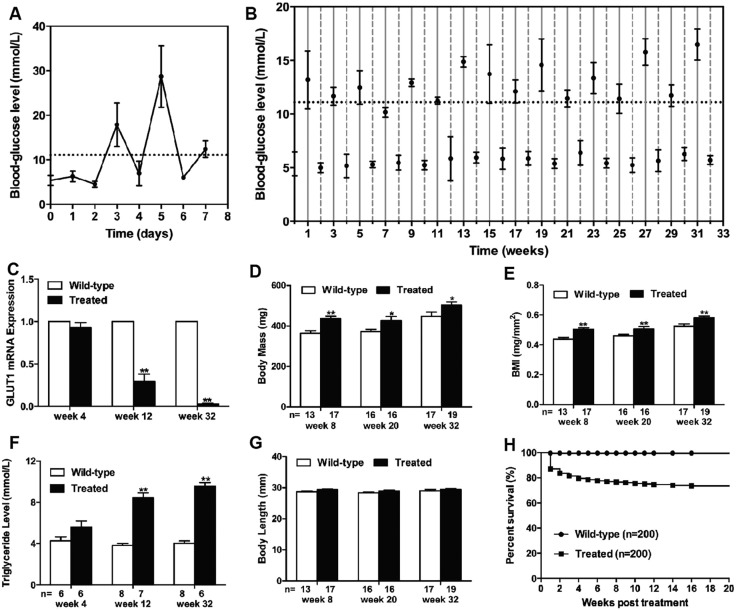
**Induction of hyperglycemia led to alterations of metabolism.** (A,B) Blood-glucose level was detected daily for the first week (A), and then weekly until 32 weeks (B). Levels fluctuated with the glucose levels in the environment. Values higher than 11.11 mmol/l (dashed line) were considered hyperglycemic. Blood-glucose levels of treated zebrafish in glucose solutions alternated between 2% (solid vertical lines) and 0% glucose (dotted vertical lines) for up to 32 weeks. (C) Downregulated expression of *GLUT1* mRNA in treated fish from week 12. (D) Increased body mass (BM) in treated fish from week 8. (E) Increased BMI in treated fish from week 8. (F) Increased triglyceride levels in treated fish from week 12. (G) Preserved body length (BL). (H) Survival curves (Kaplan–Meier representation) of the wild-type (*n*=200) and treated (*n*=200) fish within 20 weeks. Values are means±s.d. *P<0.05, **P<0.01 compared with the wild-type group. *n*, number of fish examined.

Fish blood-glucose levels fluctuated dramatically depending on the glucose concentration after treatment by alternately immersing adult zebrafish in a glucose solution or water (Fig. 1A,B). The average blood-glucose value following exposure to 2% glucose was in the hyperglycemic range, whereas it approached baseline levels following exposure to 0% glucose, which was similar to the response of diabetic individuals to dietary glucose uptake and insulin injections. Complementarily, glucose transporter-1 (*GLUT1*), which facilitates the transport of glucose across plasma membranes of mammalian cells, was detected by real-time polymerase chain reaction (PCR). *GLUT1* in cell membranes is increased by reduced glucose levels and decreased by increased glucose levels (Olson and Pessin, 1996). Hyperglycemia was accompanied by a marked reduction in *GLUT1* expression in this study (Fig. 1C), demonstrating impaired glucose utilization in the treated group from week 12. Moreover, increased body mass (BM) and body mass index (BMI) were noted in the glucose-treated fish after 8 weeks of treatment (Fig. 1D,E), which was accompanied by increasing triglyceride levels from week 12 (Fig. 1F). This finding, at least partially indicated impaired lipid metabolism. Body length (BL) was not different between the two groups throughout the whole study (Fig. 1G). Moreover, significant mortality was detected in glucose-treated zebrafish especially in the first 4 weeks (Fig. 1H).

### Hyperglycemia induced cardiac remodeling in adult zebrafish

**Fig. 2.**
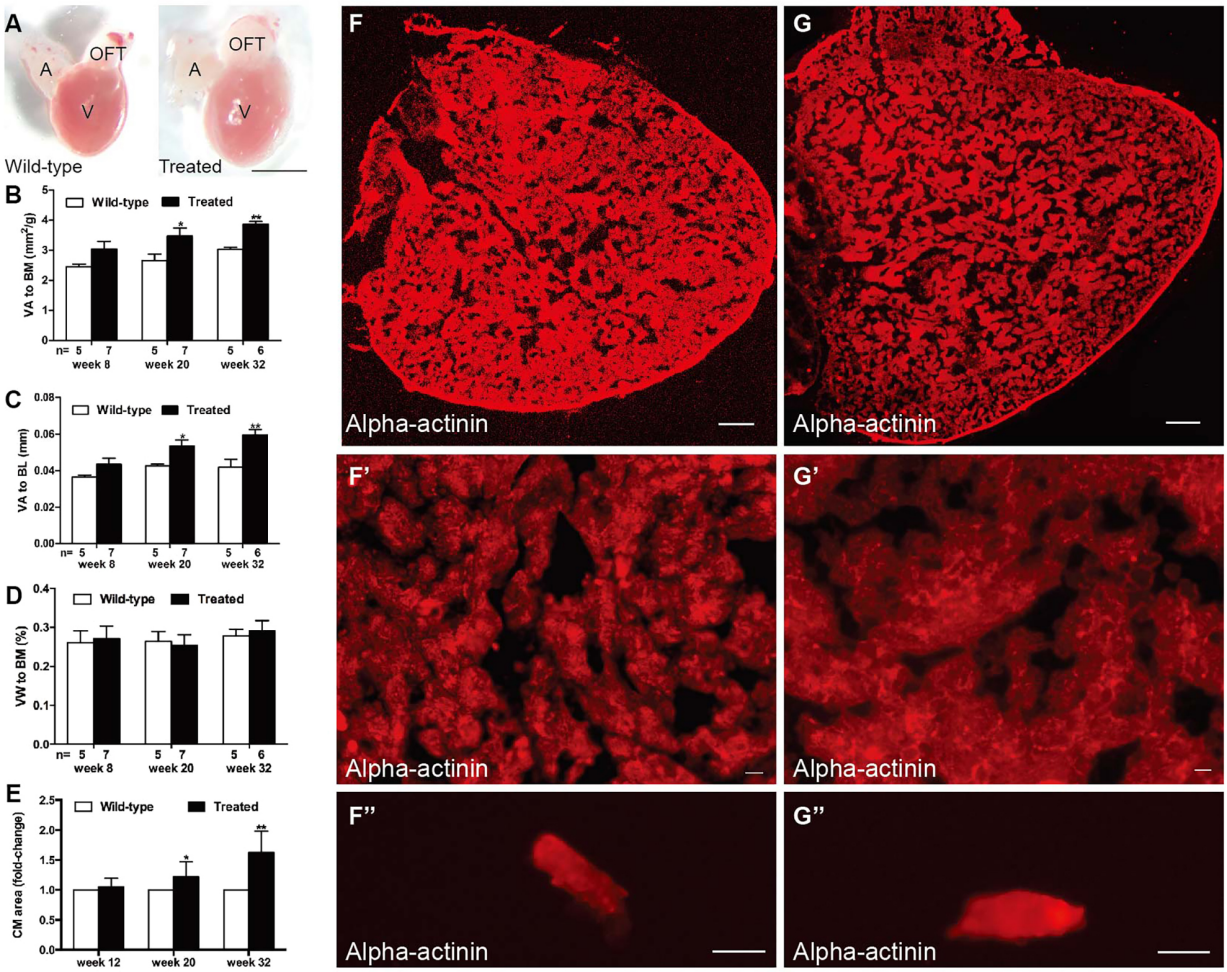
**Hyperglycemia induced heart enlargement and myocardial hypertrophy.** (A) Enlarged heart in the treated fish compared with the control group in week 20 (*n*=10 fish); scale bar: 1 mm. A, atria; OFT, outflow tract; V, ventricle. (B–D) Quantification of ventricular area to body mass (VA/BM) (B), ventricular area to body length (VA/BL) (C) and ventricular weight to body mass (VW/BM) (D) at different time points after glucose treatment. (E) Quantification of cardiomyocyte (CM) area indicated that CM size increased in the hearts of the treated fish from week 20. (F,G) Anti-alpha-actinin antibody staining showed enlarged ventricles in the treated fish (G) compared with that in the wild-type group (F) in week 32 (*n*=10 fish); scale bars: 100 μm. (F′,G′) Higher magnification images of ventricles in week 32 stained by anti-alpha-actinin antibody showed hypertrophic myocardium in the treated fish (G′) compared with that in the wild-type group (F′) (*n*=10 fish); scale bars: 10 μm. (F″,G″) Representative images of single CMs stained with anti-alpha-actinin antibody, dissociated from the hearts of the wild-type (F″) and treated (G″) fish in week 32 (*n*=10 fish); scale bars: 10 μm. Values are means±s.d. *P<0.05, **P<0.01 compared with the wild-type group. *n*, number of fish examined.

**Fig. 3.**
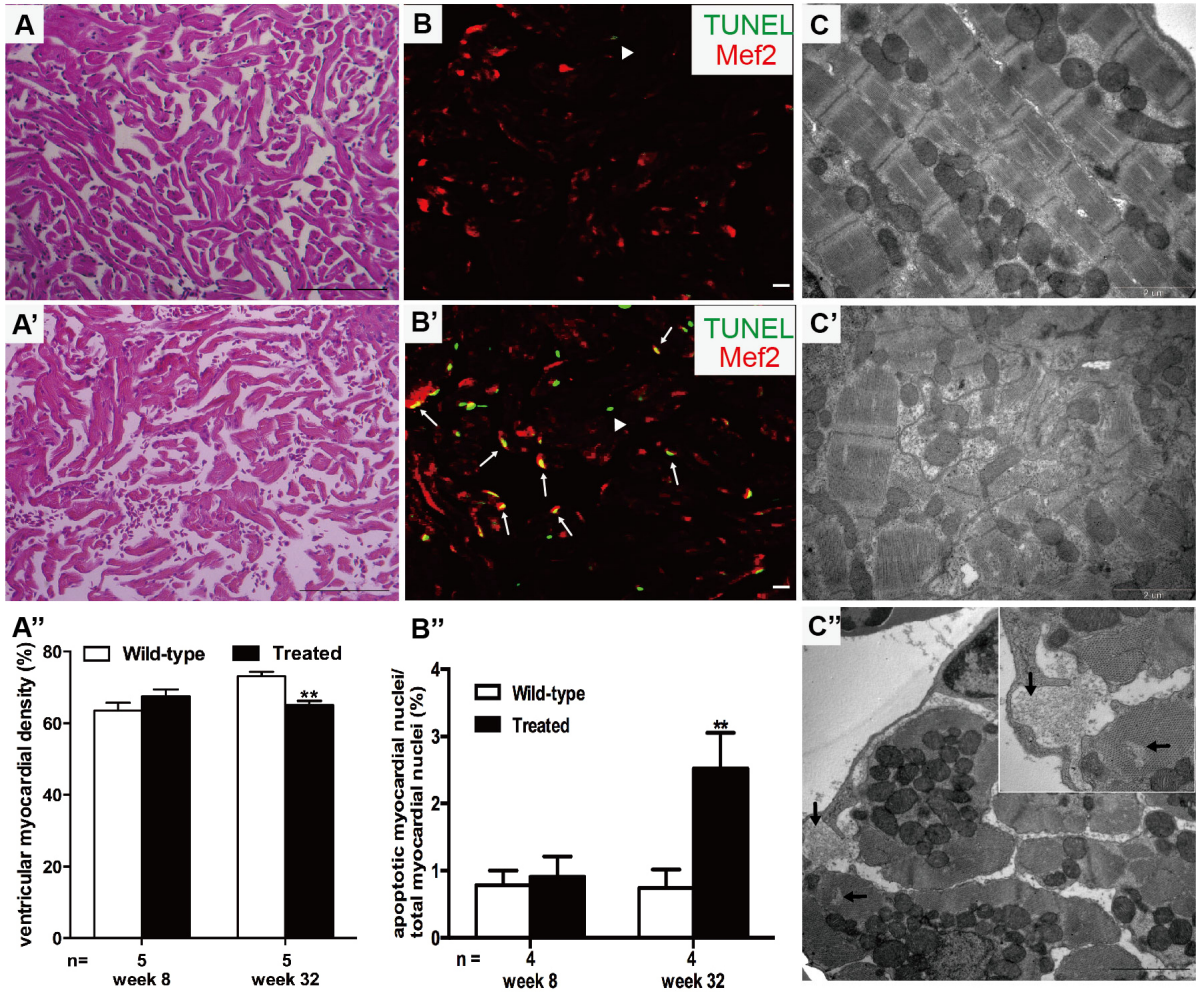
**Hyperglycemia induced muscular disarray, myofibril loss and apoptosis activation.** (A,A′) H&E staining of ventricle sections showed muscular disarray and myofibril loss in treated fish (A′) compared with the wild-type group (A) after 32 weeks of treatment (*n*=20 field, repeated five times); scale bars: 100 μm. (A″) Quantification analysis of ventricular myocardial density in H&E-stained hearts between the two groups in weeks 8 and 32. (B,B′) TUNEL (green)-stained sectioned ventricles co-stained with Mef2 (red) of wild-type (B) and treated (B′) fish in week 32 (*n*=20 field, repeated five times); scale bars: 10 μm. Arrows: TUNEL+/Mef2+; arrowheads: TUNEL+/Mef2−. (B″) Measurement of the ratio of apoptotic nuclei [yellow (green plus red)] to total myocardial nuclei (red) between the two groups in weeks 8 and 32. (C,C′) Longitudinal TEM image verified muscular disarray and myofibril loss detected in the hearts of the treated fish (C′) compared with that in the wild-type group (C) (*n*=20 field, repeated five times); scale bars: 2 μm. (C″) Transverse TEM image showed myofibril loss (arrows) in the hearts of treated fish. Inset is a higher magnification image. Scale bar: 2 μm. (A″,B″) Values are means±s.d. **P<0.01 compared with the wild-type group. *n*, number of fish examined.

Significantly enlarged hearts were noted in the hyperglycemic fish in week 20 of treatment, as evidenced by markedly higher ratios of ventricular area to body mass (VA/BM) and ventricular area to body length (VA/BL) (Fig. 2A–C). However, the ratio of ventricular weight to body mass (VW/BM) was not different between groups (Fig. 2D). Histological staining and transmission electron microscopy (TEM) were performed to further assess myocardial alterations. The examination of ventricles with the anti-sarcomeric alpha-actinin antibody (Fig. 2F–G″) suggested an enlarged heart (Fig. 2G), and hypertrophic myocardium (Fig. 2G′) and CMs (Fig. 2G″) in the treated fish in week 32. Quantification of CM area revealed increased CM size from week 20 (Fig. 2E). Hematoxylin and eosin (H&E) staining showed muscular disarray and myofibril loss in the treated fish in week 32 (Fig. 3A′) and dramatically reduced myocardial density (Fig. 3A″). Myocardial nuclei density was calculated using the number of myocardial nuclei per field to myocardial density (Fig. S1A), showing no difference in the two groups in week 32. This meant no increased number of myocardial cells in the enlarged heart. Besides, no interstitial fibrosis was detected by Masson's staining in the two groups (Fig. S1B,B′).

The ratio of apoptotic myocardial nuclei [Fig. 3B–B″, yellow (green plus red)] to total myocardial nuclei (red) was measured, indicating significant apoptosis in the hyperglycemic fish compared with the wild type in week 32. Longitudinal TEM images (Fig. 3C,C′) verified muscular disarray and myofibril loss, and transverse images (Fig. 3C″) revealed myofibril loss in the hearts of treated fish. Moreover, no significant difference was observed in proliferating cell nuclear antigen (PCNA) staining of the two groups (Fig. S1C), suggesting that myocardial hyperplasia had no effect on heart enlargement. Taken together, these results led to the conclusion that myocardial apoptosis and hypertrophy were involved in cardiac remodeling of hyperglycemic zebrafish.

### Hyperglycemia induced cardiac dysfunction in adult zebrafish

**Fig. 4.**
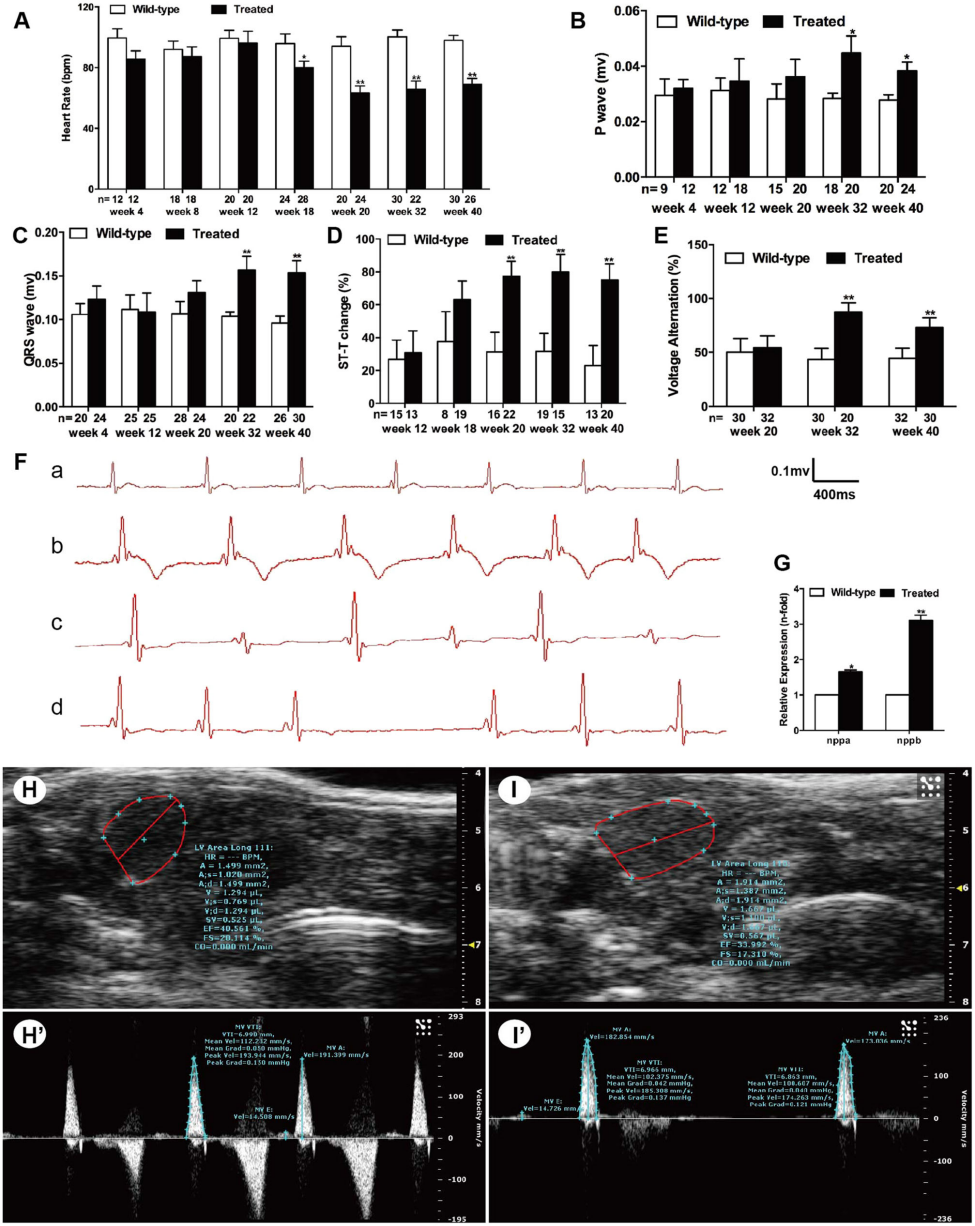
**Hyperglycemia induced cardiac dysfunction.** (A) Analysis of heart rate (HR) at different time points. (B) Analysis of P-wave amplitude at different time points. (C) Analysis of QRS-wave amplitude at different time points. (D) Percentage of ST-T change (T-wave inversion or ST-T depression). (E) Percentage of voltage alteration. (F) Typical ECG diagrams: a, normal ECG; b, T-wave inversion; c, voltage alteration; and d, prolonged PP interval (sinus arrest). (G) Real-time PCR analysis of the expression of *nppa* and *nppb* in wild-type and treated zebrafish in week 32. (H,I) Typical B-mode echocardiography images of wild-type (H) and treated (I) fish to evaluate ventricular morphology and function (*n*=20 fish). (H′,I′) Doppler-derived images of atrioventricular (AV) valve velocity of the wild-type (H′) and treated (I′) fish (*n*=20 fish). (A–F) Values are reported as means±s.d. (H–I′) Image data were automatically generated using the Vevo 2100 Workstation Software package. *P<0.05, **P<0.01 compared with the wild-type group. *n*, number of fish examined.

Electrocardiogram (ECG), performed to study hyperglycemia-induced myocardial electrophysiological changes, revealed significantly decreased heart rate (HR) from week 18 (Fig. 4A) and increased P-wave and QRS-wave amplitudes from week 32 (Figs 4B and 3C) in the hyperglycemia group. However, PR and QT intervals, adjusted by HR, showed no significant changes (data not shown). The incidence of ST-T change from week 20 markedly increased (Fig. 4D). Representative T-wave inversion is shown in Fig. 4F,b. The incremental occurrence of ‘voltage alternation’ began in week 32 in the hyperglycemic fish (Fig. 4E). A representative diagram is shown in Fig. 4F,c. Other abnormal ECG changes, such as a prolonged PP interval, were only detected in the treated fish (Fig. 4F,d). A normal ECG recorded from the untreated fish is shown in Fig. 4F,a.

Real-time PCR showed that fetal genes *nppa* and *nppb* were activated in the treated fish in week 32, with a 1.6-fold increase in *nppa* expression and 3.1-fold increase in *nppb* expression compared with the untreated fish (Fig. 4G).

Cardiac function was dynamically monitored by echocardiography to determine how long zebrafish should be treated with glucose to establish this model. No significant differences were noted in cardiac function after 12 weeks of treatment. The ventricular end-diastolic area and volume in the treated group were greater than those in the untreated group in week 20 (1.624±0.396 vs 1.231±0.321 mm^2^, *P*<0.05; 1.457±0.575 vs 0.933±0.356 μl, *P*<0.05). Isovolumic relaxation time (IVRT) adjusted by HR (IVRTa) was dramatically shortened in the treated group (6.2±3.4 vs 12.0±3.6%, *P*<0.01) and accompanied by reduced peak velocity of the atrioventricular (AV) valve (122.1±20.4 vs 155.6±32.7 mm/s, *P*<0.05).

Echocardiographic parameters differed significantly between the two groups in week 32 of treatment (Table 1). Typical B-mode echocardiography images in week 32 are shown in Fig. 4H,I. Data to evaluate ventricular morphology and function were automatically generated using the Vevo 2100 Workstation Software package (Movie 1 and 2). Representative images (Fig. 4H′,I′) were derived from Doppler echocardiography (Movie 3 and 4). The hyperglycemic fish showed a significantly increased BM. Both VA and ventricular volume (VV) increased at diastole and systole in the treated group, and, after BM correction, the marked increase was still present compared with the untreated fish. Diastolic parameters showed that the peak velocity across the AV valve was slower in the treated fish. The differences in IVRT and IVRTa between the two groups were significant. Systolic indices showed shortened ventricular ejection time (VET) adjusted by HR (VETa) and isovolumic contraction time (IVCT) adjusted by HR (IVCTa) in the hyperglycemic fish. Moreover, a markedly reduced HR and stroke volume (SV) resulted in decreased cardiac output (CO). The sharp decline in fractional area change (FAC) and ejection fraction (EF), two key indicators used to estimate cardiac function, demonstrated impaired systolic function in the hyperglycemic fish in week 32.

**Table 1.**
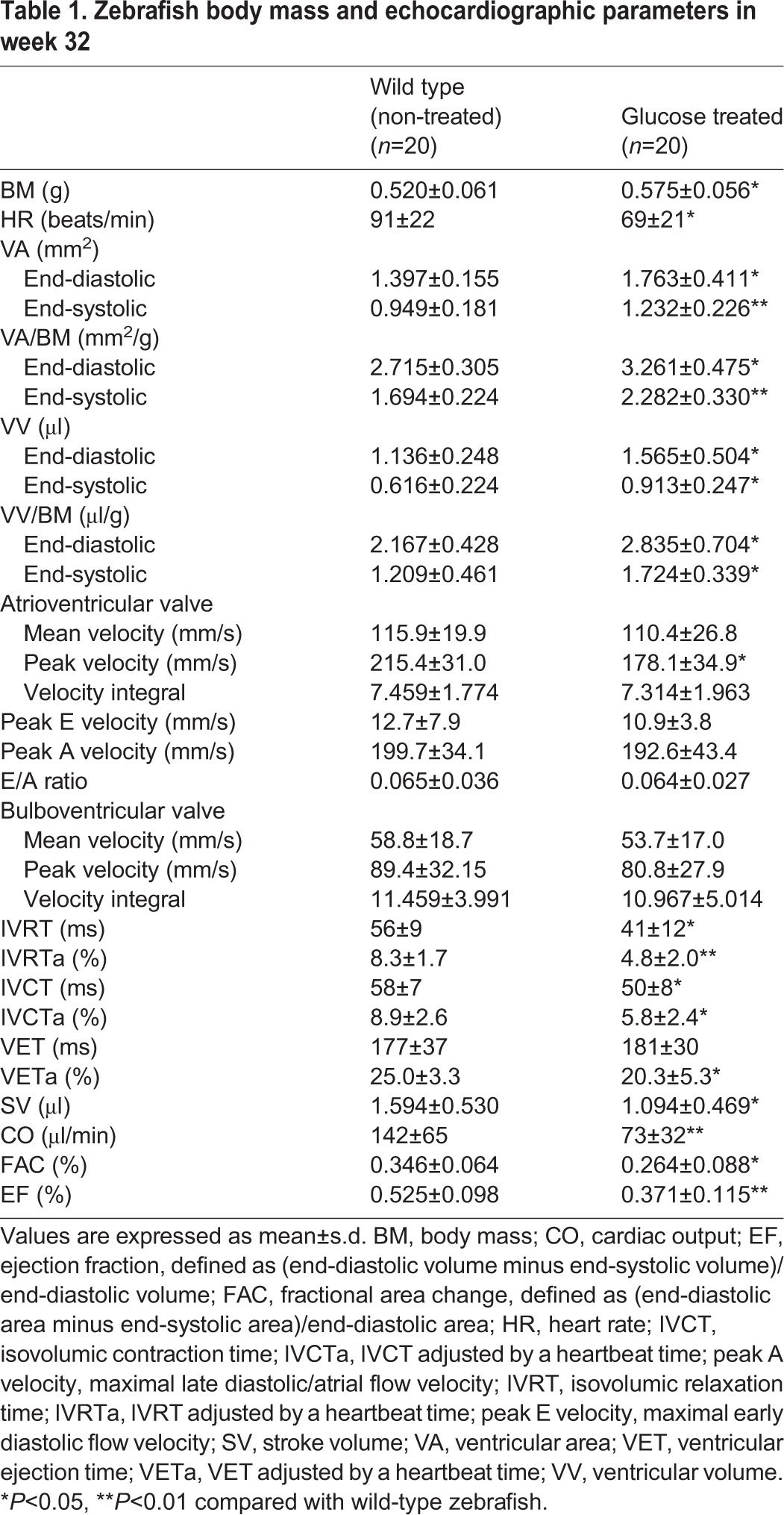
**Zebrafish body mass and echocardiographic parameters in week 32**

### Hyperglycemia induced alterations of the *Nkx2.5–Calr–p53* signaling pathway in zebrafish hearts

**Fig. 5.**
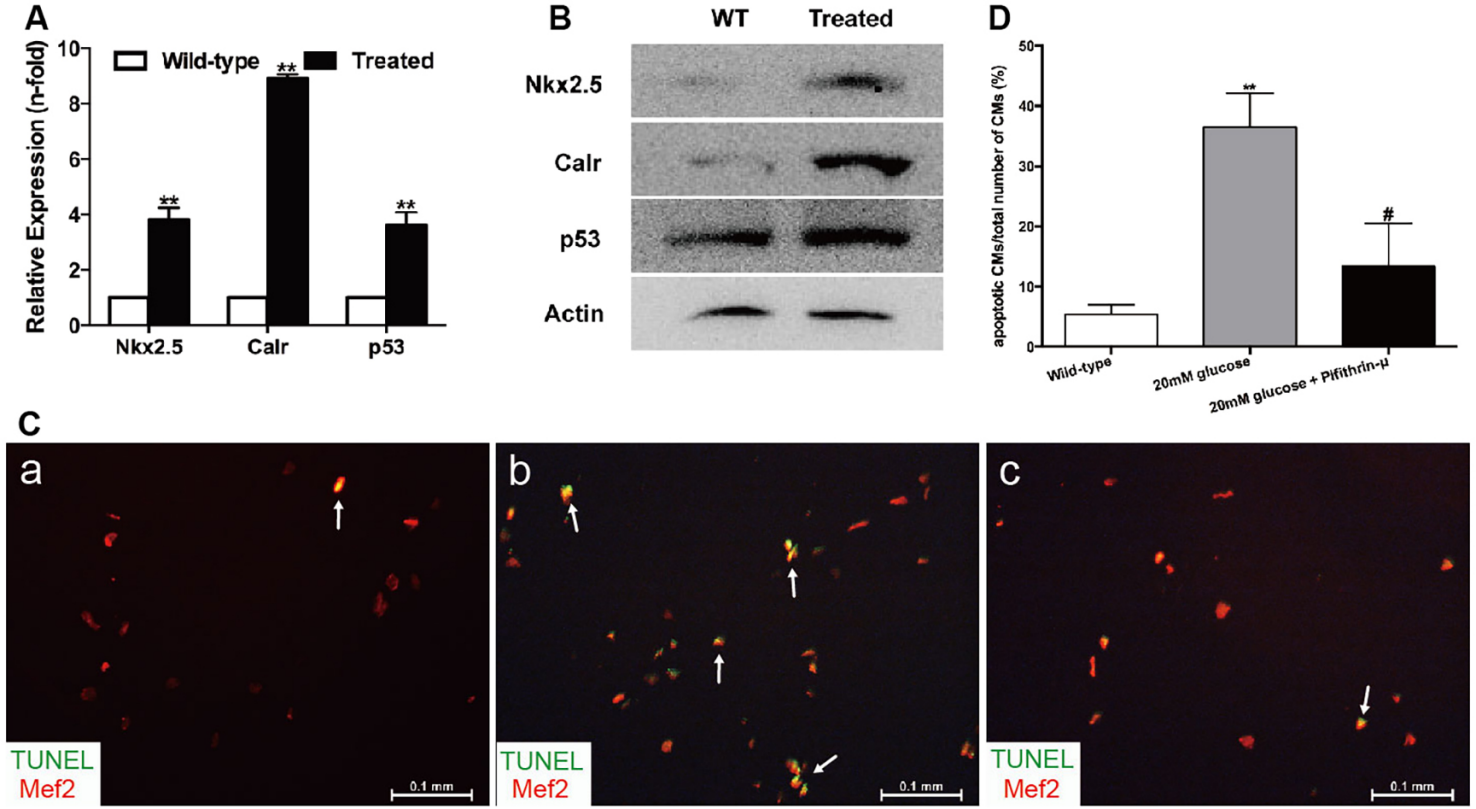
**High glucose levels induced alterations of the *Nkx2.5*–*Calr*–*p53* signaling pathway and apoptosis.** (A) Real-time PCR analysis of the expression of *Nkx2.5*, *Calr* and *p53* in the wild-type and treated fish in week 32 (*n*=8 fish per group). (B) Western blot analysis of the expression of *Nkx2.5*, *Calr* and *p53* in wild-type (WT) and treated fish in week 32 (*n*=8 fish, repeated three times). (C) Merged images of cardiomyocytes (CMs) co-stained with Mef2 (red) and TUNEL (green) in the wild-type group (a), CMs exposed to 20 mM glucose (b) and CMs exposed to 20 mM glucose together with 10 μM pifithrin-μ (c) (*n*=20 field, repeated five times). Arrows: TUNEL+/Mef2+ CMs. Scale bars: 0.1 mm. (D) Quantitation of apoptotic CMs to total number of CMs *in vitro* of the three groups: CMs without any treatment, with 20 mM glucose, or with 20 mM glucose plus 10 μM pifithrin-μ (*n*=20 field per group, repeated five times). (A,D) Bars represent means±s.e.m. **P<0.01 compared with the wild-type group. ^#^*P*<0.05 compared with 20 mM glucose group.

The expression levels of *Nkx2.5*, *Calr* and *p53* evaluated by real-time PCR were approximately 3.8-fold, 8.9-fold and 3.6-fold higher, respectively, in hyperglycemic hearts than in untreated hearts (Fig. 5A), which was consistent with the protein levels determined using western blotting (Fig. 5B). Moreover, double-staining of *Calr* or *p53* with Mef2 on cryosections of adult zebrafish hearts indicated increased expression of *Calr* or *p53* on CMs (Figs S2 and S3).

### *In vitro* high-glucose incubation of CMs induced apoptosis and alterations of the *Nkx2.5*–*Calr*–*p53* signaling pathway

**Fig. 6.**
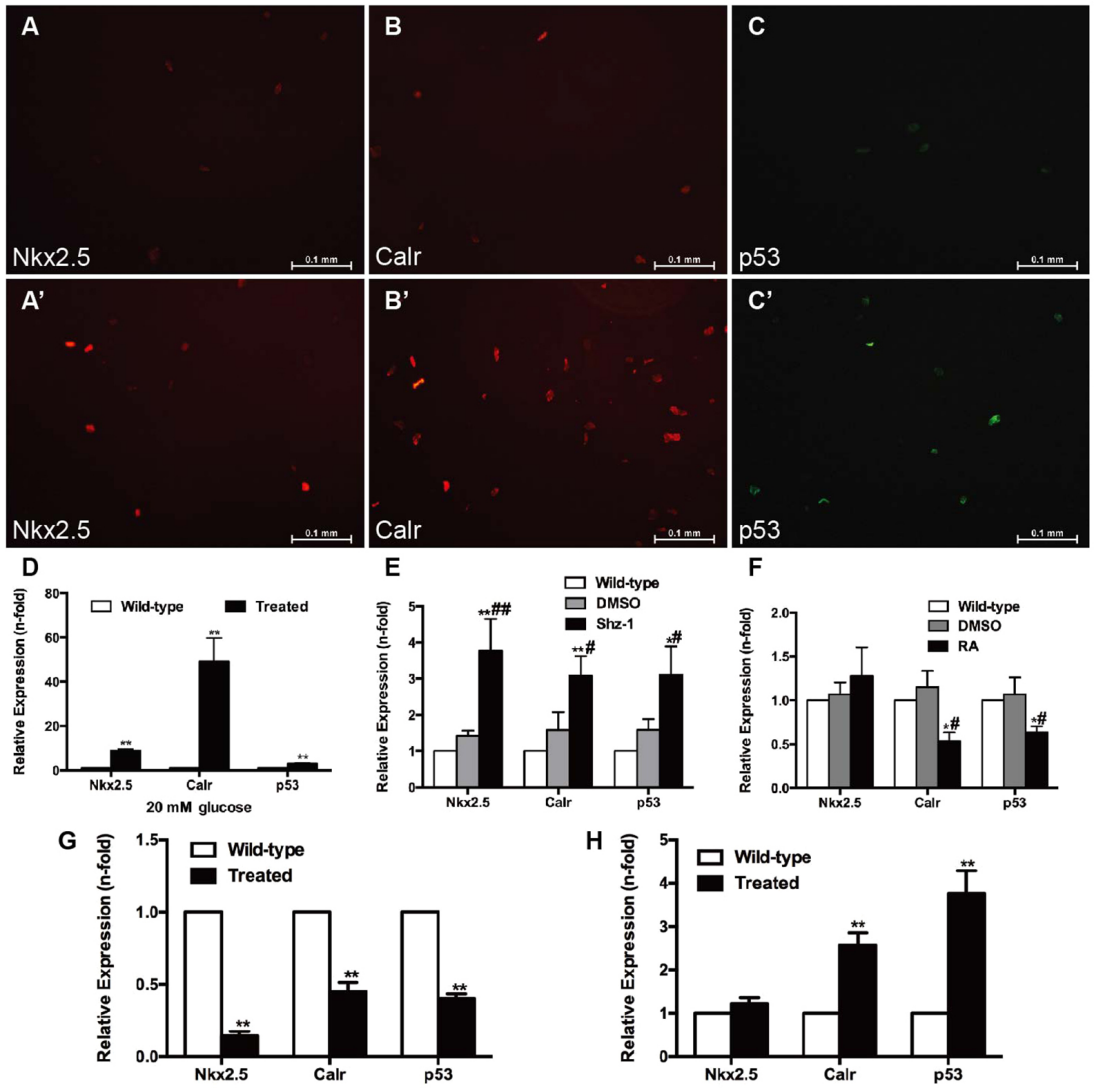
**Effects of high glucose, drugs and cell infection on cardiomyocytes.** (A–D) Cardiomyocytes (CMs) were incubated with 20 mM glucose for 24 h before analysis. (A,A′) Anti-Nkx2.5 antibody staining showing increased Nkx2.5 levels in glucose-treated CMs (A′) compared with the wild-type group (A) (*n*=20 field, repeated five times); scale bars: 0.1 mm. (B,B′) Anti-Calr antibody staining showed increased Calr levels in glucose-treated CMs (B′) compared with the wild-type group (B) (*n*=20 field, repeated five times); scale bars: 0.1 mm. (C,C′) Anti-p53 antibody staining showed increased p53 levels in glucose-treated CMs (C′) compared with the wild-type group (C) (*n*=20 field, repeated five times); scale bars: 0.1 mm. (D) Expression levels of *Nkx2.5*, *Calr* and *p53* determined by real-time PCR. (E) Real-time PCR analysis of *Nkx2.5*, *Calr* and *p53* in CMs exposed to 2.5 μM Shz-1 compared with the wild-type fish and DMSO control. (F) Real-time PCR analysis of *Nkx2.5*, *Calr* and *p53* in CMs exposed to 1 μM retinoic acid (RA) compared with the wild-type fish and DMSO control. (G) Changes in *Calr* and *p53* levels in CMs with altered expression of *Nkx2.5*. (H) Upregulation of *Calr* expression increased *p53* levels in CMs. (D–H) Bars represent means±s.e.m. (*n*=5–6 fish per group). *P<0.05, **P<0.01 compared with the wild-type group. ^#^*P*<0.05, ^##^*P*<0.01 compared with the DMSO control (0.1%).

CMs were incubated with a high-glucose medium to explore whether myocardial apoptosis was directly related to hyperglycemia. The TUNEL assay results showed that CMs incubated with 20 mM glucose for 24 h (Fig. 5C,b) exhibited increased apoptosis compared with the wild-type group (Fig. 5C,a). Incubation for 24 h with the addition of 10 μM pifithrin-μ (a specific inhibitor of p53) reduced CM apoptosis (Fig. 5C,c). Thus, apoptosis in this model could be directly attributed to hyperglycemia via p53. The ratio of apoptotic CMs to the total number of CMs is shown in Fig. 5D. The mRNA and protein expression of *Nkx2.5*, *Calr* and *p53* was consistently upregulated in CMs exposed to glucose (Fig. 6A–D). To confirm the interactions of the *Nkx2.5*–*Calr*–*p53* signaling pathway, CMs were harvested with 2.5 μM Shz-1 for 72 h and 1 μM retinoic acid (RA) for 96 h and analyzed by real-time PCR. Shz-1, used to upregulate *Nkx2.5*, activated not only *Nkx2.5* but also its downstream target genes *Calr* and *p53*, and incremental mRNA expression was detected in the hyperglycemic fish compared with the untreated fish and dimethyl sulfoxide (DMSO) controls (Fig. 6E). The addition of RA, a compound used to inhibit *Calr*, reduced the expression of *Calr* to 56% and the expression of *p53* to 63% within 96 h, but did not affect the expression of *Nkx2.5* (Fig. 6F). No differences in expression were detected compared with the untreated fish and DMSO control groups. Cell infection *in vitro* was used to inhibit the expression of *Nkx2.5* or upregulate the expression of *Calr* genetically. Downregulation of *Nkx2.5* induced decreased mRNA expression of *Nkx2.5*, *Calr* and *p53* (Fig. 6G). Upregulation of *Calr* resulted in the increased expression of *p53*, but the *Nkx2.5* level remained unchanged (Fig. 6H). Taken together, the results indicated that the *Nkx2.5–Calr–p53* signaling pathway played an important role in the development of diabetic cardiomyopathy.

## DISCUSSION

Several diabetic models such as the *db/db* mouse model and the Zucker diabetic fatty rat model have been developed to study cardiomyopathy (Bugger and Abel, 2009). An adult zebrafish model of hyperglycemia-induced cardiomyopathy was established in this study after 32 weeks of glucose treatment. Hyperglycemia in fish was accompanied by abnormal lipid metabolism, characterized by obesity and hypertriglyceridemia, which was consistent with metabolic disturbances in diabetes. The glucose-treated fish also exhibited many other similar characteristics of human diabetic cardiomyopathy.

Noninvasive echocardiography dynamically detects cardiac function *in vivo*. Diastolic dysfunction prior to systolic dysfunction was well characterized in the present model, which was consistent with the majority of evidence on diabetic cardiomyopathy (Huynh et al., 2014). Diastolic dysfunction was characterized by an enlarged ventricle, impaired IVRT and reduced peak velocity across the AV valve in the hyperglycemic fish in week 20. Echocardiographic data revealed both diastolic and systolic dysfunction in the hyperglycemic fish in week 32, and SV, CO, FAC and EF all declined dramatically, similar to what was seen in human adult diabetic cardiomyopathy. Markedly reduced IVCTa and VETa, to some extent suggested decreased myocardial contractility of hyperglycemic hearts. The development of systolic dysfunction in experimental diabetic settings might be model dependent; systolic function was preserved in FVB/N mice when using multiple low doses of streptozotocin (Huynh et al., 2010, 2013; Ritchie et al., 2012), yet decreased in streptozotocin-treated C57BL/6 mice (Westermann et al., 2007). Moreover, as illustrated in Fig. 4H′, the dramatically small maximal early diastolic flow velocity (peak E) and E/A ratio, and the relatively large maximal late diastolic/atrial flow velocity (peak A), are opposite to what is seen in humans and other higher vertebrates. Thus, assessing cardiac diastolic function via E/A ratio alteration in zebrafish has limitations.

The reduced HR seen in hyperglycemic fish was consistent with rodent models but different from what was seen in many diabetic humans (Boudina and Abel, 2007). The obvious decline in HR began in week 18, and no structural abnormalities or other functional alterations of the heart were identified at this stage. The increased frequency of ST-T change from week 20 indicated increased myocardial ischemia in the hyperglycemic fish. A previous study (Stern and Sclarowsky, 2009) reported that myocardial ischemia was more often painless in patients with diabetes (Raman and Nesto, 1996), and resting ECG abnormalities could predict silent ischemia in asymptomatic diabetic individuals (Dweck et al., 2009). Schannwell et al. (2002) proposed the viewpoint that subtle ECG alterations might be the only way to diagnose early diabetic cardiomyopathy. ECG changes occurred at a much early stage in the present fish model, which is promising for the early diagnosis of diabetic cardiomyopathy. Voltage alternation is similar to the alternating pulse commonly seen in patients with heart failure. The increased occurrence provided evidence of impaired cardiac function in hyperglycemic fish in week 32 as assessed by echocardiography. Moreover, increased P- and QRS-wave amplitudes in week 32 reflected enlarged atrium and ventricle in the hyperglycemic heart, which was verified by echocardiography. QRS-wave changes in diabetic patients have been reported, but little attention has been given to P-wave changes. Atrial enlargement usually occurs secondary to ventricular enlargement in diabetes. However, zebrafish atrium and human atria might play a different role in the cardiovascular system because, in zebrafish, the atrium has a similar size as the ventricle (Hu et al., 2001; Sun et al., 2008). Whether an early or late relationship exists between atrial enlargement and ventricular enlargement has not been shown by the present study.

Natriuretic peptides, such as NPPA and NPPB, have been considered hallmarks of increased ventricular dimensions and impaired systolic function (Raymond et al., 2003). The increased expression of *nppa* and *nppb* in the hyperglycemic fish in week 32 was another finding indicating ventricular enlargement and cardiac dysfunction.

Functional alterations are closely related to molecular and histopathological evidence of myocardial apoptosis in the hyperglycemic heart. Increased CM apoptosis is related to the transformation process from the compensated to decompensated state in the diabetic heart (Frustaci et al., 2000). Apoptosis induced by hyperglycemia caused the loss of contractile fibers and cardiac dysfunction in the present study, eventually resulting in diabetic cardiomyopathy.

The present study focused on *Nkx2.5*, *Calr* and *p53* to explore the underlying mechanism of the aforementioned findings. The increased expression of *Nkx2.5* in circulating cells was detected in patients with hypertrophic cardiomyopathy (Kontaraki et al., 2007). The significant increase in the expression of *Nkx2.5* in the hyperglycemic group in the present study suggested its potential role in hyperglycemia-induced cardiomyopathy. The expression of *Calr*, an important fetal gene, sharply declined after birth and was at a negligible level in the adult heart (Mesaeli et al., 1999). The overexpression of *Calr* in CMs promoted apoptosis (Michalak et al., 2002). *Calr* reactivation reflected impaired cardiac function in the treated fish in week 32 in the present study. Interestingly, the overexpressed *Calr* in the mouse heart induced sinus bradycardia and complete heart block (Hattori et al., 2007), indicating that *Calr* abnormalities might be associated with the dysfunction of the conduction system. The hyperglycemic fish with overexpressed *Calr* had a markedly reduced HR and prolonged PP interval; however, the detailed mechanisms by which *Calr* influenced the conduction required further study. A previous study showed that p53 was required for glucose-induced apoptosis (Keim et al., 2001) and could downregulate *GLUT1* (Schwartzenberg-Bar-Yoseph et al., 2004). The increased expression of *p53* together with apoptosis and decreased *GLUT1* were all involved in hyperglycemic cardiomyopathy, as shown in the present study.

It is important to discern whether upregulation of *Nkx2.5*, *Calr* and *p53* is a compensatory change to protect against cardiomyopathy. What is also necessary to emphasize is that hyperglycemia in the present fish model was accompanied by obesity and high triglyceride levels, which might have additive effects with hyperglycemia. Therefore, *in vitro* study of zebrafish CMs was performed to determine the direct effects of high glucose and the roles of *Nkx2.5*, *Calr* and *p53*.

First, a series of experiments were performed to explore the optimal conditions for glucose treatment of zebrafish CMs, as this had not been previously studied. Glucose concentrations of 5, 10, 20, 30, 40 and 50 mM and exposure times of 12, 24 and 48 h were studied. The results showed that exposure at a concentration from 10 to 30 mM for 24 h was the most appropriate treatment condition. Finally, treatment with 20 mM glucose for 24 h was performed in the present study. The mRNA expression of *Nkx2.5*, *Calr* and *p53* was determined when CMs were stimulated with glucose for 24 h at different concentrations, or 20 mM glucose for different times (Fig. S4). Although a previous study showed that *Nkx2.5* directly regulated the *Calr-1* promoter and activated *Calr* in rat models and humans, little was known about the regulatory effect of *Nkx2.5* and *Calr* in a fish model. Hence, CMs were incubated with Shz-1, which significantly induced the expression of *Nkx2.5*, and the expression of both *Calr* and *p53* notably increased. Genetically, knockout of *Nkx2.5* gave rise to the decreased levels of both *Calr* and *p53*. This suggested that *Nkx2.5* had a positive regulatory effect, at least on *Calr*, in the present model. One study investigated the role of *Calr* in the regulation of apoptosis via modulating *p53* expression; however, regulatory evidence of *Calr* and *p53* in the hyperglycemic heart is still scarce. RA, a *Calr* inhibitor that has been shown to reduce *Calr* in human myeloid cells by 60% within 4 days, was used in the present study (Clark et al., 2002). RA treatment led to a prominent decline in *Calr* as well as *p53* expression, indicating that reduced *p53* was directly associated with the expression of *Calr*. To support this, cell infection was performed to specifically upregulate *Calr*, leading to the increased expression of *p53*. Importantly, *Nkx2.5* was not affected by *Calr* or *p53* expression. In summary, the data suggested that *Nkx2.5* positively regulated *Calr*, and that *Calr* was also an upstream activator of *p53* and vital in the progression of hyperglycemia-induced cardiomyopathy of adult zebrafish.

In conclusion, this study indicated that an adult zebrafish model of diabetic cardiomyopathy was a novel and promising vertebrate model, complementing existing rodent models. A novel molecular pathway of *Nkx2.5*–*Calr*–*p53* was shown in hyperglycemia-induced apoptosis, which might greatly benefit future study of therapeutic interventions for diabetic cardiomyopathy.

## MATERIALS AND METHODS

### Hyperglycemic zebrafish model

Zebrafish (*D. rerio*), AB-line, 18-month-old female adults were used and divided into wild-type (*n*=200) and treated (*n*=200) groups. Fish were maintained under constant conditions of 14-h light/10-h dark at 28°C and fed twice every day. Randomly selected adult fish from different clutches (*n*=40) were placed in respective 2.5-liter tanks. The water used for wild-type and treated fish was identical (same mineral content) except for the addition of glucose. Zebrafish were exposed to freshly prepared 2%/0% glucose (Sangon Biotech Co. Ltd, Shanghai, China) solution to induce hyperglycemia. Fish were alternately placed in the two solutions every 24 h. Following alternative treatment, three fish of each group were randomly chosen for blood-glucose measurement using a blood-glucose monitor (One-Touch Ultra) every day for the first week, then every week until week 32. The procedures were performed as previously described, and glucose concentrations more than 11.11 mmol/l were considered hyperglycemic (Gleeson et al., 2007).

### Measurement of zebrafish and ventricular size

Zebrafish were anesthetized with 0.16 mg/ml tricaine (Western Chemical Industries) solution and semi-dried, and then BM in mg and BL in mm were measured. The BMI (in mg/mm^2^) was determined by BM to the square of BL for assessing obesity. VA/BM, VA/BL and VW/BM were chosen as three indexes to assess the ventricle size. The dissected hearts were placed next to a millimeter ruler and imaged using SPOT Software attached to a Zeiss Stereomicroscope to measure VA. The largest projection of a ventricle was outlined using the ImageJ software. VW (in mg) was also measured on a digital scale. VA/BM or VA/BL was then determined from the ratio of the largest projection area of the ventricle (in mm^2^) divided by BM (in g) or BL (in mm). The VW (in mg) was divided by BM (in mg) to determine VW/BM.

### Survival curve

The number of fish was counted weekly, and the total number in each group before treatment represented 100% survival. The number of fish in each group every 2 weeks was recorded.

### Triglyceride measurement

Blood samples were withdrawn from zebrafish after an overnight fast, measured using enzymatic kits (KeHua Bio-Engineering, Shanghai, China), and detected using a Multiskan MK3 instrument (Thermo Electron Corporation) at 550 nm absorbance.

### Histological staining and quantification

Paraffin sections of hearts were prepared and stained with H&E (Sangon Biotech Co. Ltd.) or Trichrome Stain Masson Kit (Baso Diagnostics Inc., Zhuhai, China) using standard protocols and then photographed using an Olympus DP70 microscope. Myocardial pixel density and myocardial nuclei density were measured on H&E sections of the entire ventricle using Adobe Photoshop 7.0 imaging software as previously published (Sun et al., 2015). The formula used was as follows: myocardial density (%)=pixel (area of myocardium)/pixel (total area of ventricle). Myocardial nuclei density=number of myocardial nuclei per field/myocardial density.

### Immunofluorescence and TUNEL assay

Cryostat-sectioned ventricles (10 μm thickness) or primary cultured CMs were incubated with primary antibody for 2 h and secondary antibody for 30 min at 37°C and imaged using a Zeiss AxioCam MRm microscope equipped with AxioVision software (Carl Zeiss). Apoptosis was detected using a TUNEL kit (Sangon Biotech Co. Ltd.) and sequentially stained with anti-Mef2 antibody to identify myocardium or CMs. For quantification, the percentages of apoptotic myocardial nuclei to total myocardial nuclei and PCNA-positive area to total myocardial area were measured in stained tissue sections, and the percentage of apoptotic CMs to total number of CMs was determined in stained CMs. Primary antibodies included those against sarcomeric alpha-actinin antibody (1:200, Abcam), Nkx2.5 (1:500, LifeSpan), Calr (1:1000, Abcam), p53 (1:200, Abcam), Mef2 (1:200, Santa Cruz Biotechnology) and PCNA (1:1000, Abcam). Secondary antibodies included Alexa Fluor 488-conjugated anti-mouse immunoglobulin G (IgG) (1:200, Abcam) and Alexa Fluor 647-conjugated anti-rabbit IgG (1:100, Invitrogen).

### Transmission electron microscopy

Dissected fish ventricles were fixed in 2% glutaraldehyde at 4°C for at least 2 h, and then dehydrated, embedded, sectioned and imaged by the Electron Microscopy Core Facility using a Philips CM-120 transmission electron microscope.

### Electrocardiogram

ECG was recorded using the BIOPAC ECG100C System (CA, USA) as previously described (Sun et al., 2015; Milan et al., 2006). Fish were anesthetized for 5 min, and each ECG was stabilized for 2 min and then recorded. ECG intervals, segments and HR were measured and statistically analyzed. ST-T depression or T-wave inversion was collectively referred to as ST-T change in the present study, suggesting myocardial ischemia in humans. The amplitude of the QRS-wave alternating between high and low was defined as voltage alternation, which was similar to the alternating pulse in humans. The prolonged PP interval in fish equated to sinus arrest in human ECG.

### Echocardiography

Echocardiography was performed as in a previous study (Sun et al., 2015). Ventricular dimensions were estimated using area (mm^2^) and volume (mm^3^), and calculated using the area–length (A–L) formula. SV (μl) and CO (μl/min) were assessed via Doppler measurement. FAC and EF were used to evaluate systolic function. FAC was defined as (end-diastolic area minus end-systolic area)/end-diastolic area. EF was defined as (end-diastolic volume minus end-systolic volume)/end-diastolic volume. Time parameters, including IVRT, VET and IVCT were calculated and adjusted by HR, representing the percentage of that of a heartbeat time, referred to as IVRTa, VETa and IVCTa, respectively. Velocity parameters such as the blood flow velocity across the AV valve and bulboventricular (BV) valve were also recorded. Each measurement was analyzed with an average of three consecutive heartbeats.

### Quantitative real-time PCR

RNA isolation and real-time PCR protocols were used as described in a previous study (Sun et al., 2015). The formula 2^−ΔΔCt^ was used to determine the fold-change in target-gene expression normalized with 18S. Gene primers are listed in Table S1.

### Western blot analysis

Dissected hearts were manually homogenized and lysed in SDS sample buffer containing protease inhibitor (Roche). Protein was extracted and subjected to western blotting following a standard protocol (Ding et al., 2011). Primary antibodies included those against Nkx2.5 (1:200, LifeSpan), Calr (1:1000, Abcam), p53 (1:200, Abcam) and beta-actin (1:5000, Sigma).

### Primary CM culture and drug treatment

CMs from ventricles were dissociated as described in a previous study (Sander et al., 2013) and cultured at 28.5°C for 24 h in L-15 media with 10% fetal bovine serum (Invitrogen) before being exposed to drugs. Shz-1 (a compound for upregulating *Nkx2.5*), RA (a compound for inhibiting *Calr*) and pifithrin-μ (a specific inhibitor of *p53*) were purchased from Sigma–Aldrich (Shanghai, China) and dissolved in DMSO for storage. Drug treatment conditions included 20 mM glucose for 24 h, 2.5 μM Shz-1 for 72 h, 1 μM RA for 96 h and 10 μM pifithrin-μ for 24 h. The final DMSO concentration was kept at 0.1%.

### Plasmid construction production of lentivirus and cell infection

The production of lentivirus and cell infection was performed as described in previous studies (Ruokun et al., 2016; Song et al., 2016). The expression of Zebrafish *Calr* plasmid was based on the lentivirus vector pCDH-CMV-MCS-EF1-Puro. First, the DNA fragment of *Calr* was obtained by PCR using the forward primer F, 5′-GACTCAGATCTCGAGATGCGGATCACTGCTGCAGT-3′ and reverse primer R, 5′-TCGACTGCAGAATTCTTACAATTCATCTTTAGGGAGCGCATCAT-3′, and cloned into the *Xho*I/*Eco*RI sites. Nkx2.5-specific and control short-hairpin RNA was constructed into the *Age*I/*Eco*RI sites of lentivirus vector pLKO.1. Short-hairpin RNA was synthesized by GenePharma (Shanghai, China): sense: 5′-GCAAAGACAGATGACACATTT-3′; antisense: 5′-AAATGTGTCATCTGTCTTTGC-3′. Shuttle vectors combined with helper plasmids were transfected into the 293T cells using Lipofectamine 2000 according to the manufacturer's instructions (Invitrogen, USA). The virus was harvested and used to infect the CMs of zebrafish.

### Statistical analysis

Continuous variables were presented as mean±s.d., and differences between two groups were compared using Student's *t*-test. Categorical variables were presented as counts or percentages, and the chi-square test was used to examine differences. Statistical significance was considered at *P*<0.05.

## Supplementary Material

Supplementary information
