# Supplementary material for: Activation of the Nkx2.5–Calr–p53 signaling pathway by hyperglycemia induces cardiac remodeling and dysfunction in adult zebrafish
Source: Dis Model Mech. 2017 Oct 1;10(10):1217–27. doi: 10.1242/dmm.026781 (PMC5665450; doi:10.1242/dmm.026781)
Supplement: Supplementary information [file dmm-10-026781-s1.pdf]

Supplementary Table 1

Table S1. Gene-specific primers for real-time PCR

Gene	Primer sequence (5'-3')
**Nkx2.5-F**	GGGATGGTAAACCGTGTCTGG
**Nkx2.5-R**	TAGTTGCTGTTGGACTGTGAAGG
**Calr-F**	AAACAGATTGACAACCCCTCCTAC
**Calr-R**	CAGCCTCCTCAACATCATCGG
**p53-F**	ATAAGAGTGGAGGGCAATCAGCGA
**p53-R**	AGTGATGATTGTGAGGATGGGCCT
**GLUT1-F**	CCTGTTGCCCTTCTGTCCTG
**GLUT1-R**	CCTCATCATCTGTCTGCTCTCG
**nppa-F**	GATGTACAAGCGCACACGTT
**nppa-R**	TCTGATGCCTCTTCTGTTGC
**nppb-F**	CATGGGTGTTTTAAAGTTTCTCC
**nppb-R**	CTTCAATATTTGCCGCCTTTAC
**18S-F**	CACTTGTCCCTCTAAGAAGTTGCA
**18S-R**	GGTTGATTCCGATAACGAACGA

F, forward; R, reverse.

Supplementary Figures

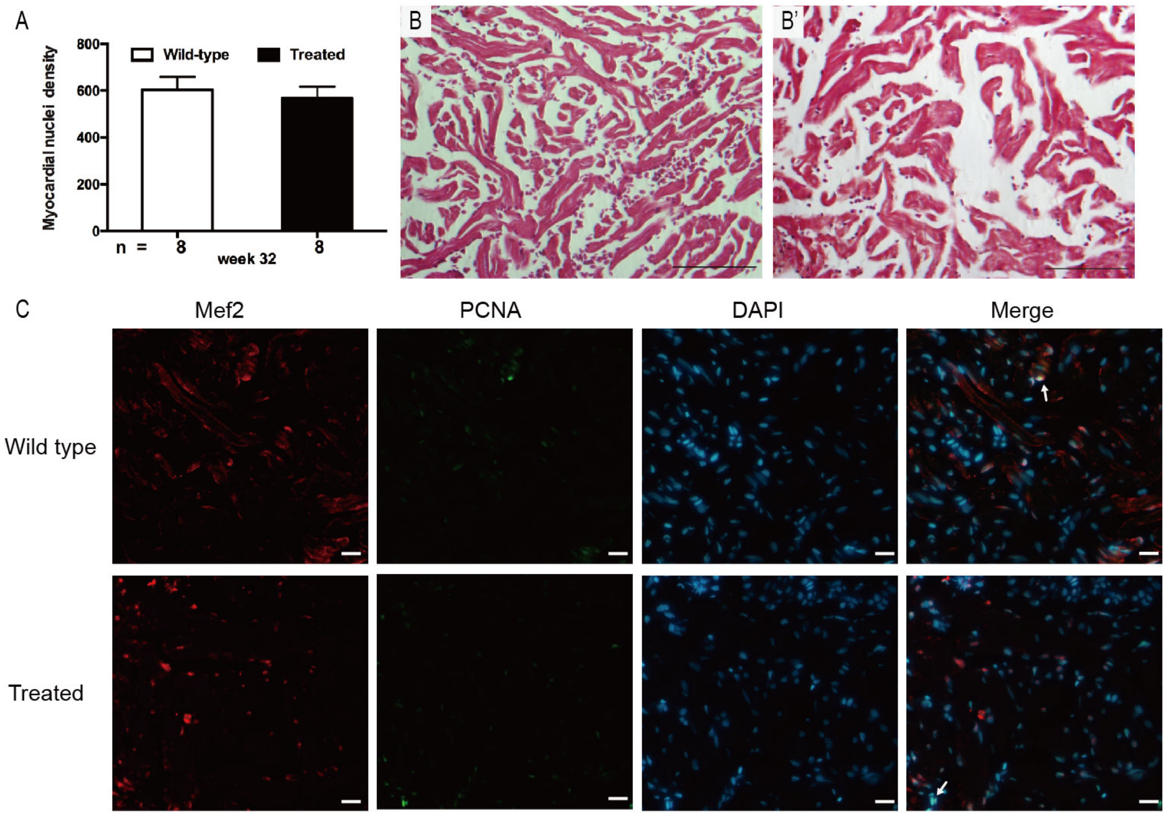

Supplementary Figure 1. (A) Myocardial nuclei density, defined as number of myocardial nuclei per field to myocardial density, showing no difference in two groups at week 32 (p > 0.05). (B) Masson's staining did not show any interstitial fibrosis in the two groups (n = 15 field, repeated five times); scale bar = 100μm. (C) Co-staining of PCNA (green), Mef2 (red) with DAPI (blue) on cryosections of adult zebrafish hearts, indicating no significant difference in PCNA staining between wild-type and treated group (*n* = 15 field, repeated five times); scale bar = 10μm； Arrows: PCNA+/Mef2+/DAPI+. (A) Bars represent mean ± standard error of the mean, n = number of fish examined.

Disease Models & Mechanisms • Supplementary information

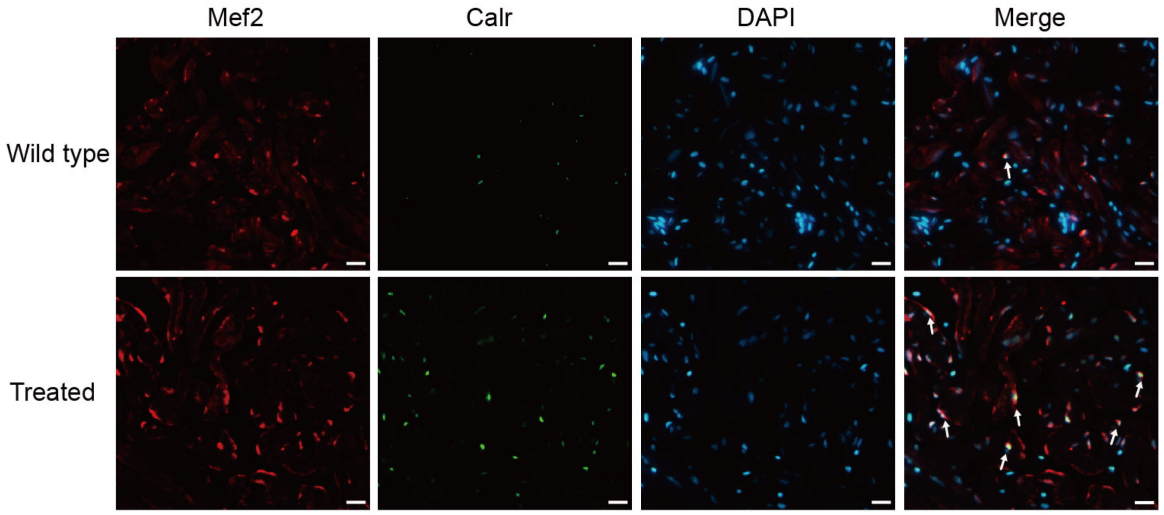

Supplementary Figure 2. Co-staining of Calr (green), Mef2 (red) with DAPI (blue) on cryosections of adult zebrafish hearts, indicating increased Calr expression on cardiomyocytes of treated fish compared with the wild-type (*n* = 15 field, repeated five times); scale bar = 10μm； Arrows: Calr+/Mef2+/DAPI+ (white, green and red plus blue).

Disease Models & Mechanisms • Supplementary information

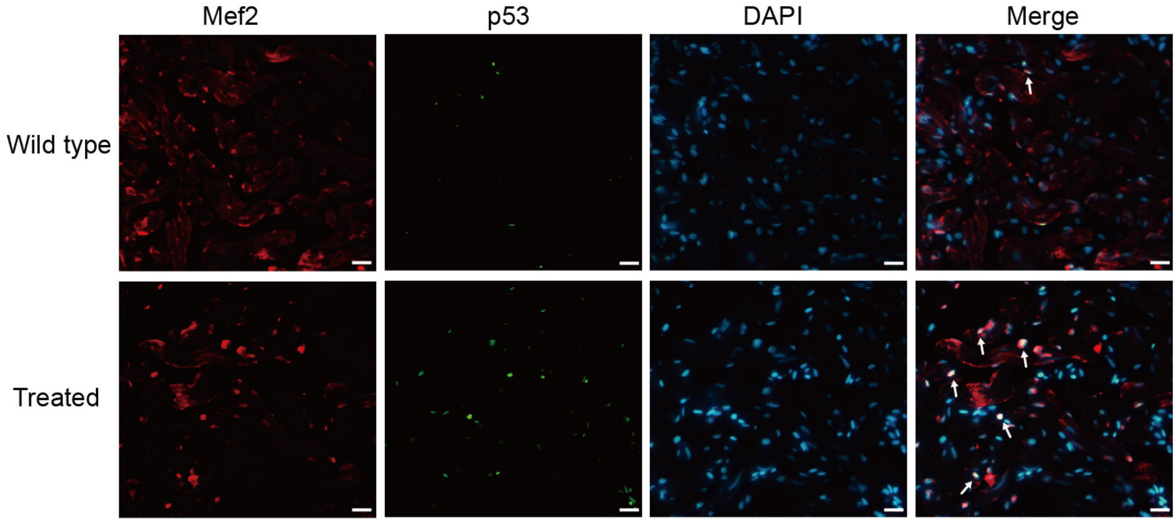

Supplementary Figure 3. Co-staining of p53 (green), Mef2 (red) with DAPI (blue) on cryosections of adult zebrafish hearts, indicating increased p53 expression on cardiomyocytes of treated fish compared with the wild-type (n = 15 field, repeated five times); scale bar = 10µm; Arrows: p53+/Mef2+/DAPI+ (white, green and red plus blue).

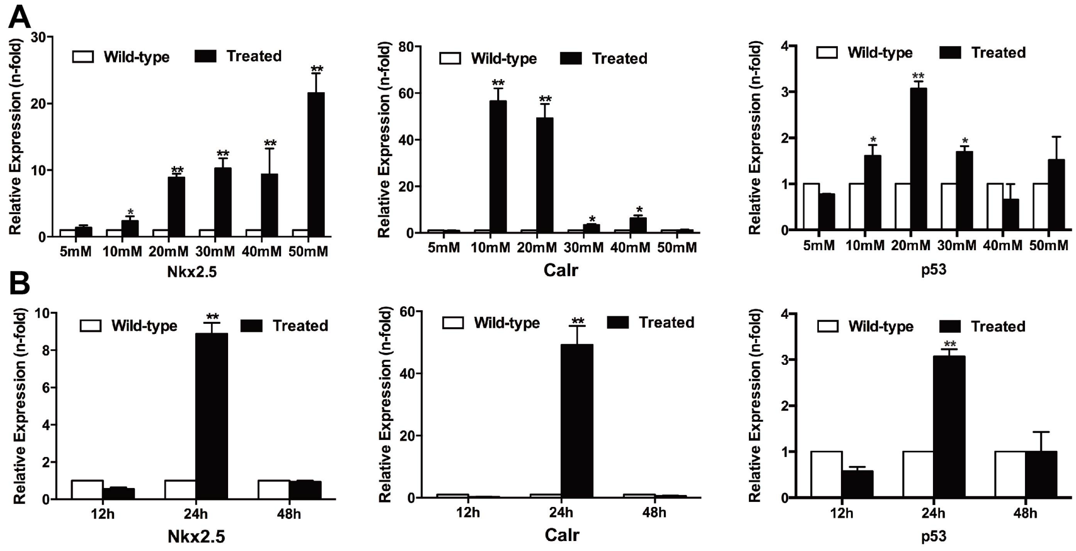

Supplementary Figure 4. Determination of the optimal conditions for glucose treatment of cardiomyocytes. (A) Real-time PCR analysis of *Nkx2.5*, calreticulin, and *p53* in cardiomyocytes (CMs) exposed to glucose for 24 h at different concentrations (5, 10, 20, 30, 40, 50 mM), compared with the wild-type group. (B) Real-time PCR analysis of *Nkx2.5*, *calreticulin*, and *p53* in CMs exposed to 20 mM glucose for different times (12, 24, 48 h). (A-B) Bars represent mean ± standard error of the mean (n = 5-6 fish per group). *$p < 0.05$, **$p < 0.01$ as compared with the wild-type group.

Disease Models & Mechanisms • Supplementary information

Supplementary Videos

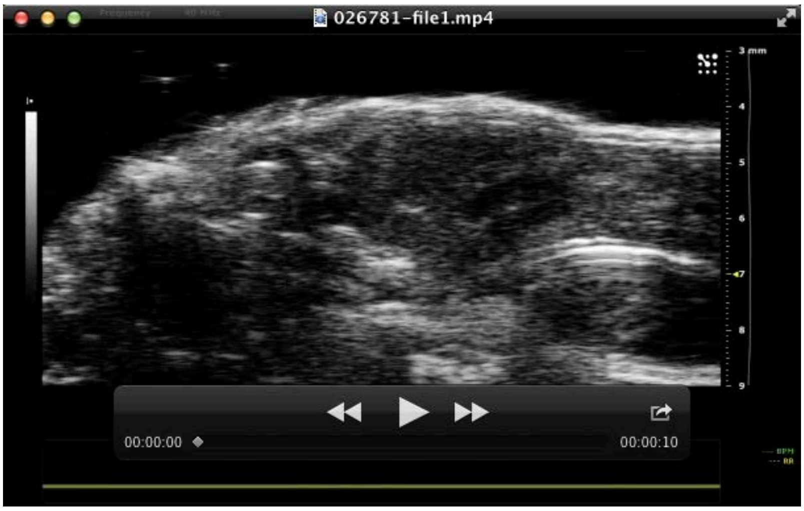

Movie 1. Ventricular morphology of the wild-type zebrafish at week 32, derived from B-mode echocardiography.

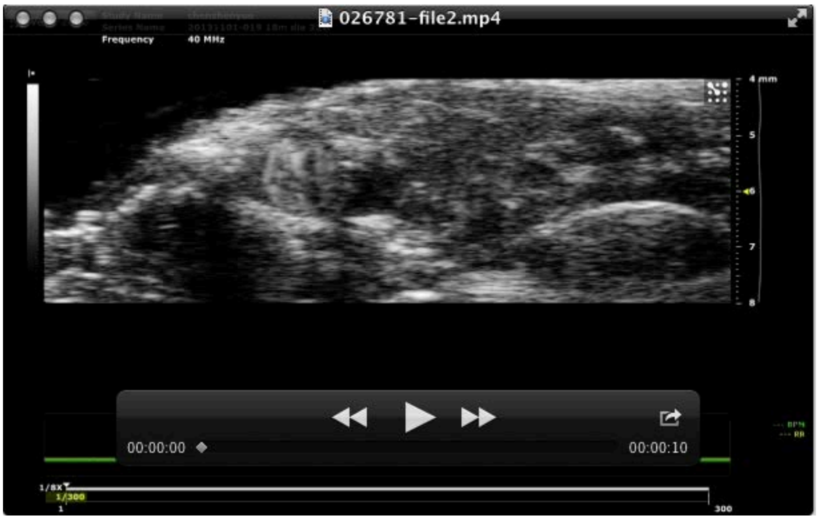

Movie 2. Ventricular morphology of the treated zebrafish at week 32, derived from B-mode echocardiography.

Disease Models & Mechanisms • Supplementary information

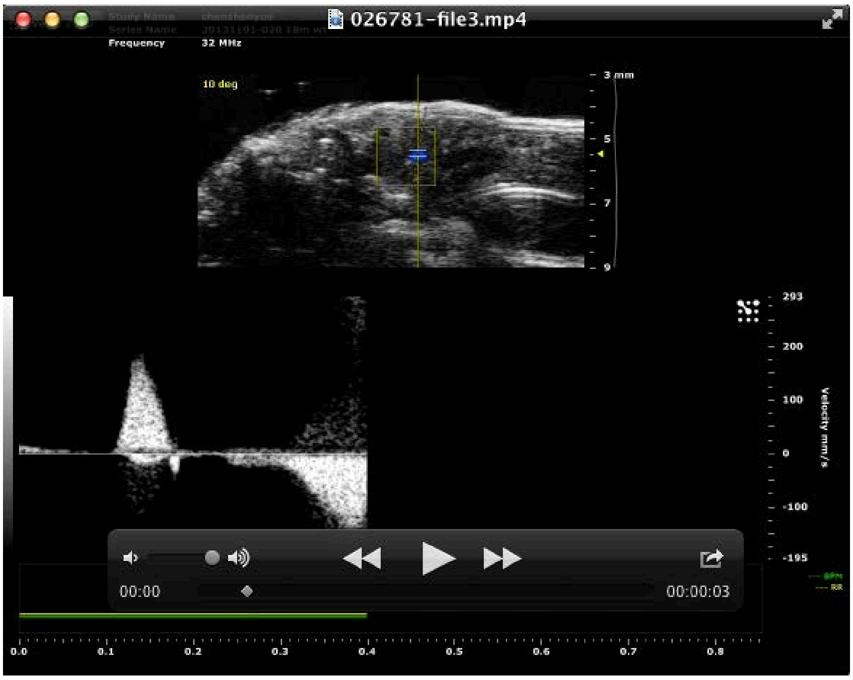

Movie 3. Atrioventricular (AV) valve velocity of the wild-type zebrafish at week 32, derived from Doppler echocardiography.

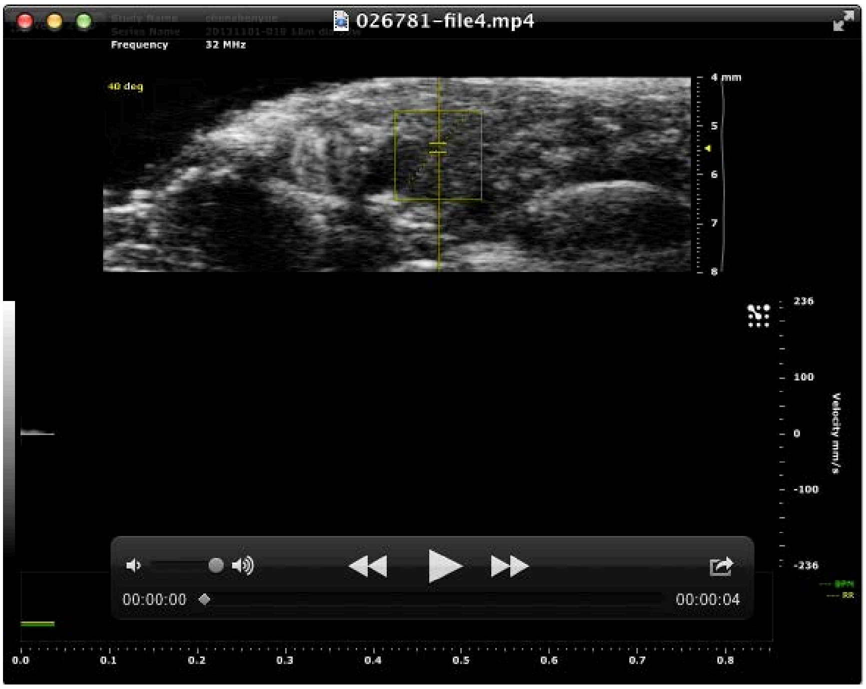

Movie 4. Atrioventricular (AV) valve velocity of the treated zebrafish at week 32, derived from Doppler echocardiography.

Disease Models & Mechanisms • Supplementary information
